# A Review of the Risk Factors and Approaches to Prevention of Post-Reperfusion Syndrome During Liver Transplantation

**DOI:** 10.1080/15476278.2024.2386730

**Published:** 2024-08-04

**Authors:** Qian Gao, Jin-Zhen Cai, He Dong

**Affiliations:** a Department of Anesthesiology, Affiliated Hospital of Qingdao University, Qingdao University, Qingdao, China; b Organ Transplant Center, Affiliated Hospital of Qingdao University, Qingdao University, Qingdao, China

**Keywords:** Ischemia, liver transplantation, reperfusion injury, review, risk factors

## Abstract

Post-reperfusion syndrome (PRS) is a severe and highly lethal syndrome that occurs after declamping the portal vein forceps during liver transplantation. It is marked by severe hemodynamic disturbances manifested by decreased mean arterial pressure, increased heart rate and elevated pulmonary artery pressure. The complex pathogenesis of PRS remains understudied. It is generally believed to be related to the large amount of acidic, cold blood that enters the circulation after release of the portal clamp. This blood is rich in oxygen-free radicals and metabolic toxins, which not only aggravate the ischemia-reperfusion injury of the liver but also further attack the systemic organs indiscriminately. Considering the range of possible adverse prognoses including acute kidney injury, delirium and graft nonfunction, it is imperative that clinicians increase their awareness and prevention of PRS. The aim of this article is to review the current risk factors, pathophysiological mechanisms and prevention strategies for PRS.

## Introduction

**Figure 1.**
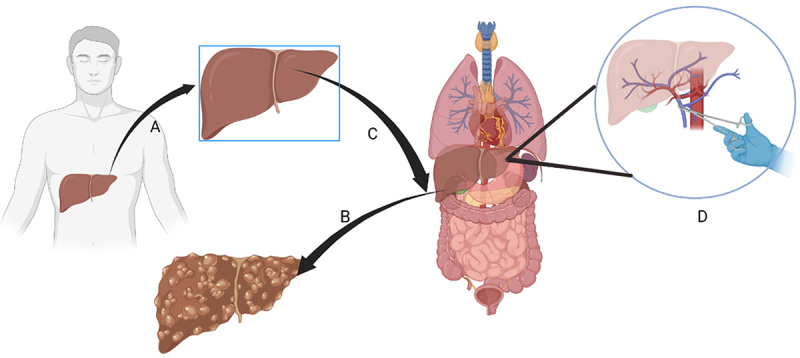
The model of liver transplantation progress. (a) Acquire liver graft from donation after cardiac death or donation after brain death then repair it. (b) Remove the deceased liver from recipient. (c) Implant the liver graft into recipient. (d) Declamping the portal vein forceps into new hepatic phase.

The surgical procedure of liver transplantation (LT) is divided into hepatic resection, anhepatic phase and neohepatic phase (Figure 1). During the neohepatic phase, especially at the moment of declamping the portal clamp, patients often present with significant hemodynamic disturbances. This phenomenon, first described by Aggarwal^1^ in 1987, referred to acute hypotension and severe heart failure that occurs after opening the portal vein clamp for graft perfusion and was defined as post-reperfusion syndrome (PRS). PRS is often accompanied by severe malignant arrhythmias or even cardiac arrest in recipients. This lethal phenomenon has attracted extensive interest from researchers. Currently, two main definitions of PRS are used, Aggarwal^1^ and Hilmi.^2^ However, the incidence^3,4^ of PRS during LT varies considerably from 3.4% to 77.4% due to differences in the definition of PRS, methods of arterial monitoring, surgical technique, source of donor liver and recipient status.

Related reasons for PRS have been explored by major research centers, focusing on surgical, donor, recipient, anesthesia and ischemia-reperfusion injury (IRI).^5^ The classical LT,^6^ steatosis liver graft^7^ and higher model for end-stage liver disease (MELD) scores^8^ in the recipient all increase the incidence of PRS. Patients with PRS generally had a poorer prognosis, as evidenced by longer intensive care unit time, delirium, acute kidney injury and a higher probability of re-operation.^9^ Considering these poor prognoses, researchers have begun to investigate preventive measures for PRS, including strategies such as ischemic preconditioning,^10^ preoperative prophylactic application of vasoactive drugs^11^ and flushing of grafts.^12^

In the present study, we not only comprehensively summarize the risk factors which presented in the study centers but also sum up all effective, novel and currently controversial prevention strategies currently proposed in major research centers. This will guide the clinic to adopt further prospective studies to continuously improve the safety and success of liver transplantation.

## Definition and clinical presentation

PRS was first described by Aggarwal^1^ in 1987 and refers to acute hypotension and severe heart failure that occurs after opening the portal vein to clamp the graft for perfusion. The current definition of PRS is not uniform; Aggarwal et al. defined PRS as a fall in mean arterial pressure (MAP) >30% of the anhepatic phase level that persists for at least 1 min, usually occurring within 5 min of liver graft reperfusion. Hilmi et al.^2^ extended the criteria for PRS by classifying the syndrome as mild and severe. Mild PRS is defined as a drop in MAP and/or heart rate (HR) of <30% of the initial level for <5 min in response to intravenous calcium chloride (1 g) and/or epinephrine (≤100 mcg) without the need for continuous infusion of vasopressors. In contrast, severe patients include those with a drop in MAP and/or heart rate greater than 30% of baseline, cardiac arrest or hemodynamically significant arrhythmia, or those who require the initiation of intraoperative vasopressor infusion. It also included prolonged (defined as lasting >30 min) or recurrent (defined as reappearing within 30 min of dissolution) fibrinolysis requiring treatment with antifibrinolytic agents.

Clearly, the definition of Hilmi et al. is more comprehensive, taking into account the entire neohepatic phase of LT, the emergence of fibrinolysis, and the efficacy of vasoactive drugs. A retrospective observational study^13^ showed that both definitions were good predictors of three-month mortality. In addition, the Aggarwal et al. definition was also considered an independent risk factor for primary graft nonfunction.

## Pathophysiology mechanism

The complex pathophysiology of PRS has not been fully elucidated, and the currently accepted mechanisms are closely related to high potassium, low calcium, hypothermia, and immediate access of acidic fluids to the recipient’s heart and great vessels.^1,5^ The hemodynamic response of recipients to allograft reperfusion is characterized by continuity and phasing: acute hypotension immediately after portal vein reperfusion, followed by a protracted phase of falling blood pressure, which continues until hepatic artery reperfusion, when the hemodynamic level off.^14^ Academic circles widely acknowledge that during the phase of hepatic artery reperfusion, the prompt elimination of the oxygen debt and acid metabolic load, which accumulate during the graft’s ischemic phase and the portal vein perfusion process, plays an essential role in sustaining the stability of vital signs.^15,16^

IRI, which plays a significant role in the progression of PRS, is an unavoidable damage to the graft. During warm ischemia time and cold ischemia time (CIT), as a result of the interruption of oxygen and blood supply, tissues are subject to damage including NA/K/ATPase dysfunction, lactate accumulation, mitochondrial inactivation and the activation of lysozymes in the hepatocytes. resulting in overproduction of reactive oxygen species (ROS). After reperfusion, large amounts of pro-inflammatory cytokines (including interleukin-1B, interleukin-2, interleukin-8 and tumor necrosis factor-α) enter the recipient’s corpuscular circulation, leading to the complement system activation and inflammatory responses. This is followed by the release of other pro-inflammatory cytokines, such as calcitonin, bradykinin, chemokines and activated complement, which are closely associated with hemodynamic instability, as manifested by PRS characterized by a fall in BP.^17^

**Figure 2.**
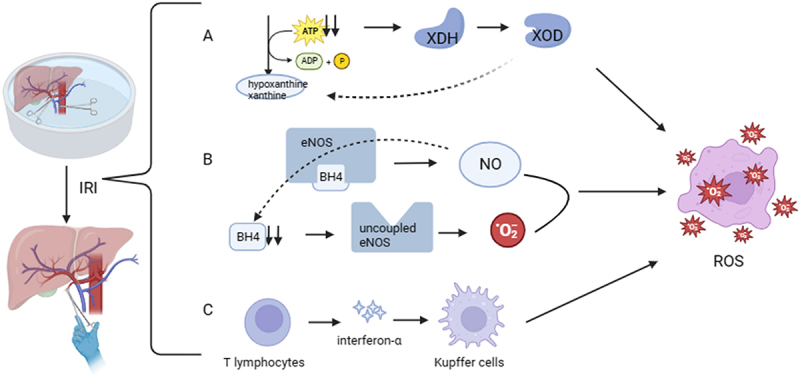
The production of ROS during IRI. (a) With the massive consumption of ATP, XDH changes into XOD. Accumulating large amounts of xanthine. (b) BH4 is heavily consumed, prompting the uncoupling of the NOS. (c) Activated Kupffer cells also promote ROS production.

The large amount of ROS produced by grafts to adapt to and resist injury is an important component of injury. During IRI, xanthine dehydrogenase is converted to xanthine/xanthine oxidase, which generates oxygen-free radicals and subsequently xanthine/xanthine oxidase produces large amounts of ROS. The cofactor of nitric oxide synthase, BH4, is greatly reduced by IRI, leading to uncoupling of nitric oxide synthase, which interferes with nitric oxide synthesis and also generates a large amount of ROS. In the early stages of reperfusion, Kupffer cells are activated, which are also involved in the production of ROS. IRI leads to activation of the graft antioxidant system as well as depletion of endogenous antioxidants and development of oxidative stress, which promotes inflammation, apoptosis and mitochondrial damage.^18,19^ Figure 2 demonstrates the generation of ROS in IRI.

After vascular endothelial cells suffer from ischemic and reperfusion injury, activated neutrophils and platelets adhere to the vascular endothelial, causing the formation of the proinflammatory and prothrombotic planes.^20^ As the endothelial cells became damaged, a series of adverse reactions occurred: increased vascular permeability, decreased BP and tissue edema. High consumption of nitric oxide and antioxidants leads to vasoconstriction thus further exacerbates the endothelial dysfunction, resulting the liver graft “no-reflow” phenomenon, graft organ dysfunction and the PRS.

Guanylate cyclase, activated by nitric oxide, catalyzes the transformation of guanosine triphosphate to cyclic guanosine monophosphate. It is now proposed that the occurrence mechanism of PRS may be related to the production of cyclic guanosine monophosphate since the effect of systemic vasodilation. There is a close relationship between the activation of cyclic guanosine monophosphate in patients with end-stage liver disease (ESLD) and hemodynamic instability during reperfusion. In addition, abnormal nitric oxide level in advanced cirrhotic patients is associated with the hyperdynamic circulatory status of the patients.^21^

## Risk factors

Summarizing the risk factors of PRS helps anesthetists to better manage LT surgery to ensure intraoperative and postoperative safety. Table 1 shows possible risk factors for PRS.

**Table 1.** The risk factors and incidence of PRS.

Year	Title	Type of study	Surgical procedure	Incidence	Risk factors	Outcome and rationale	Defination	Reference
2000	Postreperfusion syndrome in orthotopic liver transplantation.	retrospective study	OLT	12.8% (41/321)	steatotic liver grafts, long GIT, high potassium	shorten GIT as soon as possible and flush the graft	Aggarwal	[39]
2003	Causes of postreperfusion syndrome in living or cadaveric donor liver transplantations.	retrospective study	LT	48.90%	the use of greater amounts of calcium	The sudden influx of cold, acidic, hyperkalemic blood into the systemic circulation has been implicated as one of the main causes in addition to cytokine release.	Aggarwal	[50]
2007	Comparison of Femoral Arterial Blood Pressure With Radial Arterial Blood Pressure and Noninvasive Upper Arm Blood Pressure in the Reperfusion Period During Liver Transplantation	prospective study	LDLT	FABP:50.0% (18/36), RABP:80.6% (29/36), NIBP:50.0% (18/36)	/	NIBP is located closer to the cardiovascular than RABP contributing to the outcome.	Aggarwal	[22]
2008	The Impact of Postreperfusion Syndrome on Short-Term Patient and Liver Allograft Outcome in Patients Undergoing Orthotopic Liver Transplantation	retrospective study	OLT	/	/	Large transfusions not only reflect the poorer condition of the recipient, but also increase the risk of infection.	Hilmi	[2]
2009	Postreperfusion Syndrome During Liver Transplantation for Cirrhosis: Outcome and Predictors	prospective study	OLT	25% (20/75)	absence of portocaval shunt, longer CIT	IRI would alter sinusoidal endothelial then resulting hepatocyte apoptosis.	Aggarwal	[27]
2011	The occurrence of postreperfusion syndrome in orthotopic liver transplantation and its significance in terms of complications and short-term survival.	retrospective study	OLT	piggy-back technique:9.4%, classical technique:21.4%	longer CIT, classical technique.	PRS occurrence seems to be associated with higher mortality rate and worse patient outcome.	Aggarwal	[28]
2012	Incidence and predictors of post-reperfusion syndrome in living donor liver transplantation	retrospective study	LT	34.2% (58/152)	severity of liver disease, graft steatosis	Both the severity of liver disease in the recipient and graft steatosis may be risk factors for PRS in living donor LT. A	Aggarwal	[8]
2012	Postreperfusion syndrome during orthotopic liver transplantation: a single-center experience	retrospective study	OLT	16.9% (56/330)	LVDD and longer CIT	LVDD induces hemodynamic instability, leading to PRS. Longer CIT may active cellular nuclear factor kappa B and induce inflammatory responses.	Aggarwal	[47]
2013	Sympathetic withdrawal is associated with hypotension after hepatic reperfusion	retrospective study	LT	35% (77/218)	Low LF/HF and SBP	sympathova-gal imbalance and depressed SBP may be key factors predisposing to reperfusion-related severe hypotension in liver transplant recipients.	Aggarwal	[51]
2014	Hemodynamic recovery following postreperfusion syndrome in liver transplantation.	retrospective study	OLT	31.60%	older donor age, higher donor risk index, and lower central venous pressure at reperfusion.	Hemodynamic recovery after PRS appears to be phasic, frequently delayed until re-arterialization of the graft and protracted in recipients of donor grafts with higher DRI.	Hilmi	[14]
2014	Perioperative complications in liver transplantation using donation after cardiac death grafts: a propensity-matched study.	retrospective study	LT	DCD:25.7% (19/74), DBD:12.2% (18/148)	DCD	DCD grafts are prone to severe ischemia/reperfusion injury.	Aggarwal	[38]
2016	Sevoflurane Versus Desflurane on the Incidence of Postreperfusion Syndrome During Living Donor Liver Transplantation: A Randomized Controlled Trial	randomised controlled trial	LDLT	diflurane group (77.4%, 24/31) sevoflurane group (38.7%, 12/31).	the type of volatile anaesthetic	Sevoflurane alters the mechanical properties of the aorta, whereas desflurane reduces systemic vascular resistance by decreasing microarterial resistance.	Aggarwal	[3]
2018	Effects of retrograde reperfusion on the intraoperative internal environment and hemodynamics in classic orthotopic liver transplantation	prospective study	OLT	20%	/	Retrograde reperfusion may remove harmful metabolites and reduce the effects of venous return of grafts.	Aggarwal	[31]
2018	Cold Ischemia Time as a Factor in Post-transplantation Complications for Orthotopic Hepatic Transplantation. Transplantation proceedings	retrospective, observational study	OLT	CIT <6 h:3.4%, CIT >6 h:13.3%	longer CIT	CIT should be as short as possible to decrease the incidence of intra- and post-operative complications.	unknown	[4]
2019	Post-Reperfusion Syndrome in Liver Transplantation: Does a Caval Blood Flush Vent Help?	prospective observational pilot study	OLT	caval vent:19%, No caval vent:50%	/	Analysis of the initial blood flushed through the liver graft proved to be hypothermic, acidotic, and hyperkalemic.	Aggarwal	[30]
2021	Correlation between radial and femoral arterial blood pressure during reperfusion in living donor liver transplantation	retrospective study	LDLT	FABP: 52.94% (54/102), RABP:57.84% (59/102)	/	RABP nd FABP can be used interchangeably for intraoperative monitoring.	Aggarwal	[23]
2021	Hypothermic Machine Perfusion in Liver Transplantation – A Randomized Trial	multicenter, controlled trial	LT	machine-perfused liver:12% (9/72) control:27% (19/70)	/	Hypothermic oxygenated machine perfusion led to a lower incidence of PRS.	Aggarwal	[44]
2022	Influence of anesthesia type on post-reperfusion syndrome during liver transplantation a single- center retrospective study	single-center retrospective cohort study	LT	propofol:43.8% (133/304) sevoflurane:30.9%(29/94）	propofol was more strongly associated with PRS than sevoflurane	Propofol inhibits sympathetic vasoconstrictor activity and chronotropy in a dose-dependent manner.	unknown	[25]
2022	Risk factors of the postreperfusion syndrome during orthotopic liver transplantation: a clinical observational study	retrospective study	OLT	33% (400/1182)	longer CIT, classical technique.	PGNF, multiple organ failure/sepsis ，bleeding contributing the high mortality of the PRS patient.	Aggarwal	[6]
2022	Factors Associated With Postreperfusion Syndrome in Living Donor Liver Transplantation: A Retrospective Study.	retrospective study	LDLT	29.2%（73/250）	male sex，presence of small LVEDD, large GV, and long anhepatic period	male sex and presence of small LVEDD, large GV/SLV, low Ca2+ concentration and high PAP before reperfusion, and long anhepatic period were significantly associated with PRS in LDLT.	Aggarwal	[40]
2022	Hypothermic Oxygenated Machine Perfusion (HOPE) Prior to Liver Transplantation Mitigates Post-Reperfusion Syndrome and Perioperative Electrolyte Shifts.	retrospective study	LT	HOPE:12% (6/50), SCS:42% (21/50)	/	HOPE decrease the incidence of PRS may related to reducing potassium.	Aggarwal	[45]
2023	A randomized-controlled trial of ischemia-free liver transplantation for end-stage liver disease.	prospective study	LT	IFLT: 9%（3/32），CLT: 64%（21/33）	Low body temperature, hepatic release of potassium and inflammatory cytokines	ischemia-free LT would significantly reduced EAD and other complications.	Aggarwal	[76]
2023	Norepinephrine boluses for the prevention of post-reperfusion syndrome in living donor liver transplantation: A prospective, open-label, single-arm feasibility trial.	prospective study	LDLT	70%(28/40)	/	NE boluses starting with 20 μg would decrease the incidence of PRS.	Aggarwal	[93]

“/” indicates no mention in the article.

Abbreviations: OLT: Orthotopic liver transplantation, LT: Liver transplantation, LDLT: Living donor liver transplantation, GIT: Graft ischemia time, FABP: Femoral arterial blood pressure, RABP: Radial arterial blood pressure, NIBP: Noninvasive upper arm blood pressure, CIT: Cold ischemia time, IRI: Ischemia-reperfusion injury, LVDD: Left ventricular diastolic dysfunction, LF/HF: The ratio of low frequency power to high frequency power, SBP: Systolic arterial blood pressure, DCD: Donation after cardiac death, DBD: Donation after brain death, PGNF: Primary graft nonfunction, HOPE: Hypothermic oxygenated machine perfusion, SCS: Static cold storage,LVEDD: Left ventricular end-diastolic diameter, GV/SLV: Graft volume to standard liver volume ratio PAP: Pulmonary artery pressure.

### Anesthesia-related factors

When discussing risk factors for PRS, we must consider the effects of anesthetic drugs on patient physiology. Recent studies have shown that different anesthetic drugs may have a significant effect on the incidence of PRS. In a single-center retrospective cohort study under living donor liver transplantation,^22^ researchers found that the incidence of PRS was significantly lower in the sevoflurane group than in the propofol group (23.6% VS.76.4%). Jiwon Lee et al.^3^ further researched and found that the incidence of PRS in the desflurane group (77.4%, 24/31) was almost twice as high as that in the sevoflurane group (38.7%, 12/31). These differences may be related to the pharmacological actions of the anesthetic drugs: propofol inhibits sympathetic vasoconstrictor activity in a dose-dependent manner, whereas sevoflurane acts by inhibiting sympathetic activity. Diflurane reduces systemic vascular resistance by decreasing microarterial resistance.^23^ These findings suggest that the choice of anesthetic drug may be an important factor influencing the incidence of PRS.

### Surgery-related factors

The two commonly used liver transplantation techniques are classic orthotopic LT and “piggy-back” LT.^24,25^ The significant difference between them lies in whether the recipient’s inferior vena cava (IVC) is completely blocked and removed. In “piggy-back”^26^ LT, surgeons typically preserve the recipient’s IVC and perform a side-to-side anastomosis with the donor’s IVC, maintaining continuous blood flow during transplantation and reducing hemodynamic changes. It can not only lower the patient’s cardiac load and venous pressure but also mitigate IRI to the liver. Although “piggy-back” technique may be associated with outflow obstruction, Budd-Chiari syndrome and other complication,^27^ the classical orthotopic LT is considered as an important independent risk factor^28^ for PRS due to frequent hemodynamic disturbances and severe IRI.^6^

Compared with the technology of flushing the vena cava without portal vein, the portal vein flushing reperfusion technique without venting the vena cava lead to a smoother hemodynamic state and earlier graft functional recovery.^29^ Further study^30^ has analyzed that on the basis of the portal vent and a chilled LR/albumin portal flush solution, pre-reperfusion venting of vein cava appears to remain a more stable hemodynamic. Avoiding using the portocaval shunt would also decrease the incidence of PRS.

Furthermore, the various sequence of reperfusion may affect the incidence of PRS. Yang et al.^31^ found that the incidence of PRS was reduced to 20% in patients undergoing retrograde inferior vena cava reperfusion, which may attribute to the low-pressure venous blood. It not only avoids the explosive release of oxygen-free radicals but also removes the harmful metabolites in the donor liver in time, reducing the disturbance of acid-base, electrolyte as well as the violent hemodynamics fluctuations after reperfusion. In a prospective nonrandomized study,^15^ data demonstrated that the initial hepatic artery reperfusion results in a slower increase in oxygen consumption, and a less rapid increase in carbon dioxide elimination, than portal vein reperfusion. In a retrospective study,^32^ patients with simultaneous hepatic artery and portal vein reperfusion showed a better outcome than initial portal vein or hepatic artery reperfusion (Overall survival was 8.1 vs. 4.8 vs. 5.9 y). In animal experiments, the initial hepatic artery reperfusion group was also found to exhibit more severe IRI.^33^ Although the hepatic artery delivers oxygenated blood and recovery of hepatic oxygenation is accelerated by initial hepatic arterial reperfusion. However, the initial hepatic artery reperfusion does not include the primary hepatic blood supply, i.e., portal venous blood flow and may leave the liver in a state of relative ischemia for a period of time until portal reperfusion is restored.^34^ This approach may increase the risk of IRI, PRS and other postoperative complications in the recipient.^35,36^ It is certain that each reperfusion order has its own value in specific clinical situations. In short, further study about reperfusion sequence is needed.

### Donor-related factors

The graft condition is the key factor in determining the success of the liver transplantation.

Grafts from older,^37^ cardiac death,^38^ steatosis donor^39^ and large volume graft^40^ are high-risk grafts since containing more vasoactive factors. The incidence of PRS is significantly higher in patients receiving liver graft from donation after cardiac death (25.7%,19/74) than donation after brain death (12.3%,18/148).^38^ For donor livers with moderate macrosteatosis, massive fat would accumulate in the liver cells, leading to an increase in cell volume which may lead hepatic sinusoids partial or complete obstruction. When the hepatic microcirculation is impaired, its tolerance to IRI may be reduced.^39^

CIT, from the start of donor cryopreservation to the reperfusion of liver graft, was considered as an independent risk factor for developing PRS.^4^ Donor warm ischemia time, the period from the cessation of the donor’s heartbeat to the removal of the liver, is currently considered a risk factor for the development of PRS.^41^ Shortening recipient’ warm ischemia time which is defined as the time from the donor liver leave the preservation solution to the perfusion of the liver graft would offset the adverse effects of prolonged CIT.^42^

PRS occurs less frequently in recipients who receive mechanically perfused livers. Hypothermic Oxygenated Machine Perfusion has been proved to decrease the incidence of PRS since it can wash the harmful substances and metabolic waste in the donor liver as well as provide sufficient oxygen to restore its metabolic process.^43–45^ In addition, Researchers found the preservation solution also related to PRS. The high incidence of PRS in University of Wisconsin (UW) solution may be attributed to the high potassium component than the Celsior solution (21.6% vs 5.9%).^34^ Another retrospective study^46^ showed that the incidence of PRS was higher in the histidine–tryptophan–ketoglutarate solution than the UW one (61.3% vs 28.6%.). Histidine in the histidine–tryptophan–ketoglutarate solution would convert to the vasodilator histamine, which leads to longer transient vasodilatation, resulting in a higher incidence of PRS.

### Recipient-related factors

Experimental and clinical studies have proved that liver failure significantly affects cardiac function. Previous review has proposed that left ventricular dysfunction which causing hemodynamic instability is a major risk factor for the occurrence of PRS.^47^ The obvious correlation between model for MELD score and the incidence of PRS has been proved.^8^ Patients who encounter PRS always have higher MELD scores, and even a higher MELD score can independently predict the incidence of PRS in patients with fulminant liver failure.^48^ Hyponatremia (preoperative sodium <130 mmol/l) was also recognized as a predictor of PRS.^6^ Hyponatremia,^49^ including hypovolemic or hypervolemic (dilutional) hyponatremia, occurs in approximately 22% of patients with cirrhosis. Correct recipients’ electrolyte disorder properly may be an effective strategy to decrease the incidence of PRS.

Central venous pressure, as an indicator of blood volume, is concerned by anesthesiologist. Lower central venous pressure is considered as risk factors of PRS.^50,51^ After unclamping the portal forceps, 18–30 ml/100 g blood would flow into the graft. If the blood volume is insufficient, it will lead to the reduction of the preload, affecting the recipients’ clinical monitoring indicators, eventually the PRS emerged.^52^

The renin-angiotensin-aldosterone system is the main endocrine system that regulates BP, modulates the constrictive activity of blood vessels as well as sustains blood volume. Cirrhosis patients’ cardiovascular system cannot respond to vasoconstrictor substances sensitively, leading to the excess accumulation of circulating vasodilators and cardiodepressive substances which result in hyperdynamic circulatory syndrome. Higher HR at the beginning of the operation has been seen as the risk factor of PRS. Therefore, the increased HR not only reflects the hyperdynamic state of the heart but also indicates the severe state of liver and the occurrence of PRS. In addition, researchers also suppose that PRS rate could be higher in hemodynamically unstable patients who had a greater need for pressor amines, and therefore resulting in higher HR.^53^

## Intraoperative blood transfusion

One of the most important functions of the liver is to produce a large number of coagulation factors to maintain the balance between the coagulation, anticoagulation and fibrinolysis systems. However, the ESLD patients are in decompensation state, leading to the defect of their coagulation function and substantial blood loss. In the anhepatic stage, the synthesis of coagulation factors is reduced, while tissue-type plasminogen activator raise and plasminogen activator inhibitor-1 keep unchanged, leading to fibrinolysis.^54,55^ After reperfusion, the donor liver releases a large amount of endogenous heparin to aggravate the patients’ coagulation dysfunction.

It has been noted that patients with severe PRS require more intraoperative transfusions of blood products than patients without PRS or those with only mild PRS.^56^ In addition, Chung IS et al.^8^ suggested that massive blood transfusion prior to reperfusion may lead to an inflammatory response causing the release of large quantities of vasoactive substances, thereby promoting the development of PRS. Especially, during reperfusion stage, massive blood transfusion can lead to hypothermia and electrolyte disturbances that may cause aspects of hemodynamic disturbances in PRS. At the same time, massive transfusions dilute platelets and coagulation factors, affecting the coagulation status of the patient’s blood and potentially exacerbating the fibrinolytic manifestations of PRS.^2^ Literature^9,57^ also pointed out that the transfusion of blood transfusion may influence the survival and prognosis of patients undergoing LT. This observation has also been confirmed in pediatric LT.^58^

## Prognosis

PRS is now considered to be potentially inextricably linked to the patient’s prognosis. Patients are subjected to intraoperative resuscitation due to a dramatic drop in BP and severe malignant arrhythmias, and there are precedents of unsuccessful resuscitation and even death.^47^

By analyzing the prognosis of patients who underwent LT, we found that the incidence of patients presenting with postoperative acute kidney injury was generally high (12%–80%). Previous studies have suggested that the development of postoperative impairment of renal function is associated with higher preoperative MELD scores, preexisting renal function problems, and higher BMI in patients.^59^

Nowadays, it has been found that the occurrence of PRS during LT may also lead to postoperative renal dysfunction in patients.^6,28,47,60^ The associated pathogenesis is not precise. Investigators believe that in patients with intraoperative PRS, a sustained drop or maintenance of low BP stimulates renal vasoconstriction. The renal function status of patients with ESLD is strongly correlated with the severity of liver disease. Especially in patients with advanced cirrhosis, infections, gastrointestinal bleeding, nephrotoxic drugs, and diuretics often lead to inadequate renal perfusion, which further exacerbates renal ischemic-hypoxic injury due to the thickening of the capillary wall in cirrhotic patients.^28^ At the same time, high doses and concentrations of vasoactive drugs are used to maintain BP. This will further constrict blood vessels, exacerbating renal ischemic-hypoxic injury and leading to necrosis of renal tubular cells. Some researchers^17^ also believe that after the unclamping of the portal forceps, the blood carries a large amount of inflammatory mediators, acids, and ROS from the graft into the circulation. These cytokines indiscriminately attack organs throughout the body, causing a systemic inflammatory response that further involves the kidneys. In severe cases, this effect can lead to perioperative death, which is one of the main reasons for the high mortality rate in patients undergoing PRS. However, in a recent literature,^61^ researchers conducted a multifactorial analysis, finding that PRS was not an independent risk factor for the development of acute kidney injury. This may be related to different criteria for defining acute kidney injury, the condition of the recipient, the allocation of the donor, etc.

Certain research centers have found that patients who experience PRS intraoperatively are more likely to experience complications such as reoperation, delirium, and graft immune rejection.^6^ Reoperation is most often due to postoperative hemorrhage, and in the definition of Himili, fibrinolytic time duration has also become a measure of the severity of PRS. Delirium, as fluctuating changes in a patient’s consciousness. and attention, is more common in patients undergoing PRS since the prolonged mechanical ventilation, prolonged hospitalization, hyponatremia, and infections.^6,62^ Peak bilirubin levels are often applied to respond to the functional status of the graft, and it was found that patients with intraoperative PRS generally had higher peak bilirubin levels than non-PRS patients at 5 days postoperatively.^8^ The clinical manifestation of PRS is a dramatic drop in blood pressure after reperfusion, and this hemodynamic fluctuation may disrupt the microcirculation of the graft, thereby affecting graft flow and function. PRS has a direct pathophysiological link to IRI, which may increase the risk of graft dysfunction. Exposure of grafts to IRI leads to the release of inflammatory mediators and activation of the immune response, exacerbating tissue damage and graft rejection through a cascade effect.^63,64^ In animal studies,^65^ reduction of IRI was found to reduce acute immune rejection in mice. In addition, it has been pointed out that the coagulation system is an important component of the body’s immunity, and that the coagulation and fibrinolytic systems play an important role in graft IRI and graft rejection.^66^ Therefore, there is a close relationship between the occurrence of PRS and immune rejection of grafts. Table 2 shows the prognosis for patients with PRS.

**Table 2.** Prognosis of patients undergoing PRS.

Title	Year	Incidence of PRS	Postoperative renal dysfunction	Other complications	Conclusion	Refer.
Analysis of postrevascularization syndrome after orthotopic liver transplantation: the experience of an Australian liver transplantation center	2001	Aggarwal definition PRS:29/100 Control group:71/100	Serum creatinine levels were significantly higher in the PRS group on day 7.	There were no significant differences between the two groups in terms of rejection rate, biliary complications, hepatic artery thrombosis, initial graft malfunction, and survival.	PRS did not significantly affect postoperative complications or survival of patients.	[32]
The Impact of Postreperfusion Syndrome on Short-Term Patient and Liver Allograft Outcome in Patients Undergoing Orthotopic Liver Transplantation	2008	Hilmi definition No/mild:152/338 Significant 186/338	unmentioned	Patients in PRS group not only had longer time for ventilator, ICU and hospital, but also had a higher incidence of retransplantation(3.3% vs 8.6%).	The severity of PRS correlates with the prognosis of liver transplant recipients and the status of the graft.	[2]
Postreperfusion Syndrome During Liver Transplantation for Cirrhosis: Outcome and Predictors	2009	Aggarwal definition PRS(-):20/75 PRS(+):55/75	Patients who experienced PRS had a higher incidence of postoperative renal failure(70% vs 36%)	Patients who experienced PRS had a lower early survival(80% vs 96%)and a higher early ICU death (2% vs 4%).	Strong association between the occurrence of intraoperative PRS and postoperative renal failure and early postoperative mortality	[26]
Hyperdynamic Circulation in Acute Liver Failure: Reperfusion Syndrome and Outcome Following Liver Transplantation	2010	Aggarwal definition PRS(-):24/58 PRS(+):34/58	unmentioned	Patients who developed PSR experienced higher in-hospital mortality(33% vs 11%)	The occurrence of intraoperative PRS is strongly associated with postoperative mortality in patients.	[42]
Incidence and predictors of post-reperfusion syndrome in living donor liver transplantation	2012	Aggarwal definition PRS: 58/152 Non-PRS:94/152	unmentioned	Peak bilirubin (an indicator for early graft dysfunction) was significantly higher in the PRS group in the first 5 days after LT.	Further prospective studies on the relationship between PRS and post-transplant outcome are needed.	[8]
Postreperfusion syndrome during orthotopic liver transplantation: a single-center experience	2012	Aggarwal definition PRS:56/330 non-PRS:274/330	The PRS group had a higher incidence of renal dysfunction (19.23% vs 8.4%).	Patients in PRS group also had higher intraoperative (7.14% vs 0%) and postoperative mortalities (26.92% vs 12.04%).	The occurrence of intraoperative PRS is strongly associated with early poor patient prognosis.	[41]
Post-reperfusion syndrome during orthotopic liver transplantation, which definition best predicts postoperative graft failure and recipient mortality?	2017	Aggarwal definition PRS(+):351/794 PRS(−):443/794 Hilmi definition No/mild:639/794 Significant 155/794	unmentioned	The 3-month mortality, PGNF or 31 d mortality and PGNF or 3 m mortality were significantly different between the PRS and non-PRS groups under both definitions.(*p* < 0.05)	PRS has been shown to be an independent risk factor for post-transplant mortality and the development of PGNF.	[13]
The Postreperfusion Syndrome Is Associated With Acute Kidney Injury Following Donation After Brain Death Liver Transplantation	2017	Aggarwal definition PRS:53/155 nonPRS:102/155	AKI:61/155	/	The occurrence of PRS was significantly associated with the development of postoperative AKI.	[52]
Risk factors of the postreperfusion syndrome during orthotopic liver transplantation: a clinical observational study	2022	Aggarwal definition PRS(−):782/1182 PRS(+):400/1182	A significantly higher proportion of recipients in the PRS group developed postoperative renal dysfunction requiring treatment with RRT (18% versus 3.2%).	Patients in PRS group would experienced more delirium (10.5% vs 5.1%), major cardiovascular event (11.5% vs 2.6%) and longer ICU time. The mortality rate (27.25% vs 5.11%) and postoperative surgical revision （25% vs 6.6%）in the PRS group were also higher.	There is an association between the occurrence of PRS and poor postoperative liver transplantation outcomes and patient prognosis.	[6]

“/” indicates no mention in the article.

Abbreviations: PRS: Post-reperfusion syndrome, LT: Liver transplantation, PGNF: Primary graft nonfunction, AKI: Acute kidney injury, ICU: Intensive care unit.

## Prevention approaches

Considering the aforementioned series of postoperative complications of LT due to the dramatic hemodynamic fluctuations and massive inflammatory mediator release of PRS, it is urgent to find effective methods to reduce the incidence of PRS as soon as possible. By reducing the incidence of PRS, it is possible to maintain smooth hemodynamics in patients, stable blood perfusion of the graft and other organs, and thus improve the prognosis of patients undergoing LT. Meanwhile, the prevention of massive bleeding triggered by prolonged fibrinolysis and the possible concomitant immune rejection will further optimize the prospects for post-operative recovery in liver transplant patients.

### Anesthetic medication and monitoring

#### Internal environment homeostasis

Hyponatremia is an electrolyte disorder which is commonly seen in patients with ESLD. Some researchers believe that the serum sodium level below 130 mmol/l is an independent risk factor for the occurrence of PRS.^6^ Regular monitoring and timely correction of hyponatremia are essential to mitigate the risk of PRS, while also avoiding osmotic demyelination syndrome in hypernatremic patients.^67^ After the release of the portal clamp, hyperkalemia poses a significant threat to life, necessitating prompt regulation of serum potassium levels with calcium chloride and insulin.^68^ After the release of the portal vein forceps, moderate correction of acidemia without excessive use of sodium bicarbonate is advised to maintain hemodynamic stability.^69^ These integrated measures are instrumental in enhancing the success rate of liver transplant surgeries and optimizing patient outcomes, with ongoing research set to further elucidate the link between electrolyte imbalances and PRS to inform clinical decision-making.

#### Choice of anesthetic drugs

Little is known about the effects of anesthetics on PRS. Emerging evidence^25^ suggests that sevoflurane may attenuate PRS occurrence due to its cardioprotective properties, although definitive conclusions await results from larger-scale studies comparing sevoflurane to desflurane across diverse liver transplantation contexts. Propofol, an intravenous anesthetic similar to α-tocopherol, is thought to counteract oxidative stress and IRI. Propofol may improve the recovery of graft function and microcirculation by attenuating the inflammatory response, down-regulating the MMP-9 response and increasing the expression of heme oxygenase-1. However, its antioxidant and anti-inflammatory effects have not been effectively demonstrated in clinical studies.^25,70^ These preliminary findings provide a theoretical basis for the potential role of propofol in PRS prevention.

### Improvement of surgical programmes

Ischemic preconditioning

Ischemic preconditioning (IP) is a surgical strategy to protect graft from IRI through multiple, transient, mild ischemia and reperfusion management. IP as a non-lethal injury that has been shown to trigger endogenous protective mechanisms in different organs.

An experiment^71^ which was conducted in porcine using three different IP methods showed that IP strategy brings no significant benefits. Limitation factors in this experiment, including the small sample size and the short CIT, may affect the creditability of the result. Clinical findings showed a significant decrease in aspartate aminotransferase levels on postoperative day 1 and maximum aspartate aminotransferase levels at postoperative week 1 in recipients receiving pretreated grafts. Remote ischemic preconditioning acts by inhibiting inflammatory responses and activating various hepatoprotective subcellular populations through interactions between neural, humoral, and systemic pathways.^10^ Interestingly, clinical experience^72^ shows that this strategy has no obvious effect on either the quality of the graft or the prognosis of IP patients. Patients receiving an ischemic pretreated graft may have even more fatal outcomes.

There is still no conclusion on whether IP will reduce the incidence of PRS and improve the patient outcome. Therefore, the clinical research on IP is still ongoing.

#### Ischemia-free liver transplantation

The concept of ischemia-free organ transplantation is currently emerging in clinical practice.^73^ It differs from conventional mechanical perfusion in that this approach allows for uninterrupted perfusion of the graft during donor acquisition, preservation, and implantation. The graft is protected from the IRI that occurs when the blood supply is completely interrupted for a period of time and then reintroduced. The feasibility and safety of ischemia-free organ transplantation has been demonstrated in heart, liver, and kidney transplantation.

In the first study related to ischemia-free liver transplantation published in 2017, researchers^74^ found that the recipient had low levels of inflammatory mediators and no postoperative complications such as vascular, biliary, or graft rejection. Later, the investigators^75,76^ further found that this approach also significantly reduced the incidence of intraoperative PRS, which also had a positive prognosis for postoperative patients. Patients who underwent ischemia-free liver transplantation generally had a lower incidence of PNF and EAD, faster recovery of liver function, and relatively shorter postoperative intensive care unit time. Especially for fatty liver donor livers, the safety of the procedure is further guaranteed with the application of ischemia-free liver transplantation. In the next step, research centers should vigorously study more advantages of ischemia-free liver transplantation and then promote and implement this technique on the basis of safety and feasibility.

### Donor-related precautions

#### Washout technique

The relationship between PRS and hyperkalemia has been discussed.^1^ Preservation solutions usually contain high levels of potassium, and after release of the portal vein, large amounts of potassium ions enter the circulation, leading to the development of PRS and hyperkalemia. Compared with patients who underwent anterograde reperfusion only, patients with assisted irrigation of donor liver had a lower incidence of PRS. The same is true for living donor liver transplantation.^77,78^

Currently, many healthcare providers use albumin solutions to flush liver grafts through intravenous access. Researchers^79^ observed that 500 ml of 5% albumin solution flushed out 90.8% of potassium. Studies of flushing solutions remain popular. Researchers^80^ have even concluded that flushing solution potassium above 6.75 mmol/L is associated with severe PRS after reperfusion. The method of reperfusion also affects the recipient’s intraoperative hemodynamic stability and postoperative outcome.^46^

#### Donor selection and preservation

To bolster the efficacy of liver transplants and mitigate the risk of PRS, stringent donor selection and optimal graft preservation are essential. Donor selection should ideally prioritize the exclusion of high-risk grafts derived from elderly individuals, donors after cardiac death, or those with steatotic livers. These factors are correlated with elevated levels of vasoactive mediators and a heightened susceptibility to postoperative morbidity.^37,39^

In contemporary practice, the static cold storage of liver grafts has been complemented by emerging preservation modalities including normothermic machine perfusion,^81^ hypothermic oxygenated machine perfusion^45^ and abdominal normothermic regional perfusion^82^ which exhibit distinct advantages. These innovative preservation strategies are posited to attenuate cellular metabolic activity and oxygen consumption, thereby mitigating the activation of the complement cascade and dampening immune responses. Consequently, these methods have been associated with a marked reduction in the incidence of postreperfusion syndrome, enhancing the overall efficacy and safety of liver transplantation procedures.

### Receptor pre-delivery

#### Protease inhibitors

Studies have shown that the intraoperative application of aprotinin was able to reduce the vasopressor requirements effectively during early reperfusion stage.^83^

By acting on Kallikrein-Kinin system, high dose of protease inhibitors can stabilize vascular tone, reduce the release of vasoactive substances and inflammatory factors and maintain hemodynamic stability. Recently, a research center concluded that the applying of aprotinin could not prevent or alleviate the PRS.^84^

Epsilon-aminocaproic acid, an antifibrinolytic agent, has been shown to be effective in stabilizing hemodynamics and reducing blood transfusion.^85^ However, it is worth noting that the use of antifibrinolytic agent would lead to the formation of intracardiac thrombosis, causing worse outcomes.^86^

#### Magnesium sulphate

Magnesium is an essential mineral element for the human body, involved in various life activities including immune regulation, coagulation process and so on. Magnesium has been found to reduce graft injury during ischemia and reperfusion through mechanisms such as stabilizing cellular transmembrane potential, reducing calcium influx and reducing cellular energy requirements.^87^ Magnesium sulfate, an important cofactor in the coagulation cascade, was shown to improve coagulation factor activity in a randomized clinical trial study. This certainly contributes to correcting the coagulation profile in patients with end-stage liver disease.^88^

Chung et al.^89^ found that intraoperative administration of magnesium sulfate 35 mg/kg reduced the incidence of PRS and shortened the duration of PRS in patients. The researchers concluded that the higher levels of IL-4 and IL-10 in the magnesium sulfate pre-treated group were related to the fact that magnesium sulfate promotes the transfer of T-cell activity to Th2 lymphocytes, which produce anti-inflammatory cytokines that help to maintain a better cellular homeostasis. The B-cells are also driven to differentiate and produce immunoglobulins to further maintain the balance of immune levels. This will maintain homeostasis, prevent inflammatory storms and cascade reactions, thereby reducing the incidence of PRS.

However, in a recent study,^90^ it was not concluded that pre-application of magnesium sulfate reduces the incidence of PRS. This may be related to the surgical method, quality of donor liver, severity of liver disease and other factors. This controversial conclusion suggests the need for further exploration and research.

#### Mannitol

Mannitol, which also acts as an oxygen radical scavenger, has also attracted interest. However, the role of mannitol in reducing the incidence of PRS during LT is still controversial. In a double-blind randomized controlled trial,^91^ investigators found that after unclamping the portal forceps, patients who infused with 1 g/kg mannitol during anhepatic phase had higher MAP, cardiac output, and central venous oxygen saturation than the untreated group.^92^ Undoubtedly, the incidence of PRS was also significantly lower in the mannitol group.

Researchers attribute this phenomenon to the scavenging effect of mannitol on oxygen-free radicals. However, in a study^92^ conducted by Moataz Maher Emara et al. on living donor liver transplantation, it was found that mannitol did not significantly reduce the incidence of PRS. This may be related to intraoperative fluid management of patients and the specificity of living donor liver transplantation itself. Therefore, the role of mannitol on PRS still needs to be further explored.

#### Vasoactive drug

In an effort to reverse the current passive situation of emergency medication administration after the onset of PRS, clinicians are currently exploring strategies for prophylactic medication administration prior to the reperfusion of the graft, including the use of norepinephrine, terlipressin and isoproterenol. Currently, there is inconsistency among research centers regarding prophylactic medications to reduce the incidence and severity of PRS. Ho-Geol Ryu et al.^11^ conducted a prospective study and found that prophylactic application of either 10ug epinephrine or 100ug phenylephrine prior to graft perfusion reduced the incidence of PRS compared to the control group (*p* < 0.006). A recent study^93^ has indicated that initiating norepinephrine boluses at a dose of 20 µg is both viable and efficacious in reducing the likelihood of PRS during liver transplantation from living donors. Investigators^94^ have noted that the dose of target-controlled infused isoproterenol may be associated with early graft function after LT. Terlipressin, a drug has been proven to reduce the risk of hepatorenal syndrome,^95^ was also found to reduce the severity of PRS in a recent study.^96^ In addition, we need to be aware of the possible side effects of terlipressin such as increased pulmonary artery pressure. Prospective studies with appropriate sample sizes are needed to investigate the correlation between these variables. However, it is imperative to acknowledge that the administration of vasoconstrictors may exert a prophylactic effect on postoperative complications associated with PRS, while concurrently risking the underestimation of the actual incidence of PRS.^97^

### Monitoring hemodynamics

#### Blood pressure monitoring

To avert PRS, meticulous hemodynamic monitoring is essential. Studies^23^ in living donor liver transplantation confirm the reliability of both radial and femoral arterial pressures for intraoperative PRS risk assessment (52.94% vs 57.84%). However, patient-specific factors and interventions like vasopressors and volume resuscitation can influence blood pressure readings. Thus, careful site selection for blood pressure monitoring is critical, along with close monitoring of blood pressure and other hemodynamic indices to detect early signs of PRS and implement preventive actions.

#### Transesophageal echocardiography

To prevent PRS, comprehensive hemodynamic monitoring is critical. Central venous pressure may be less sensitive due to positioning and surgery, making transesophageal echocardiography essential for real-time blood volume tracking and cardiac ejection function.^98,99^ Transesophageal echocardiography also aids in detecting air embolism and thrombi, crucial for liver transplant safety. During surgery, transesophageal echocardiography, combined with other parameters, allows for precise fluid and drug management, forming a key strategy to reduce PRS risk.^98,99^

## Future directions

Considering the high incidence and high dangerousness of PRS, clinicians can build a predictive model based on the risk factors for PRS summarized in this article. Shift the focus forward and adopt active prevention strategies for patients with high-risk factors for PRS to keep their risk within a manageable range. As the prognosis of patients undergoing LT is now gradually becoming a hot research topic and a focus of clinical attention. Considering the intraoperative dangers and postoperative complications associated with PRS, it is urgent for clinicians to actively research preventive strategies and coping strategies for PRS. Moreover, despite the current high number of confounding factors, clinicians should also remain focused on further exploring preventive strategies for decreasing the incidence of PRS. In this article, we summarize in detail the novel, feasible, and controversial preventive strategies proposed by various research centers, which provide a solid foundation for further prospective clinical studies.
